# Supplementary material for: Proton gradients and pH oscillations emerge from heat flow at the microscale
Source: Nat Commun. 2017 Dec 1;8:1897. doi: 10.1038/s41467-017-02065-3 (PMC5711904; doi:10.1038/s41467-017-02065-3)
Supplement: Supplementary file 1 — Supplementary Information [file 41467_2017_2065_MOESM1_ESM.pdf]

Supplementary Figures

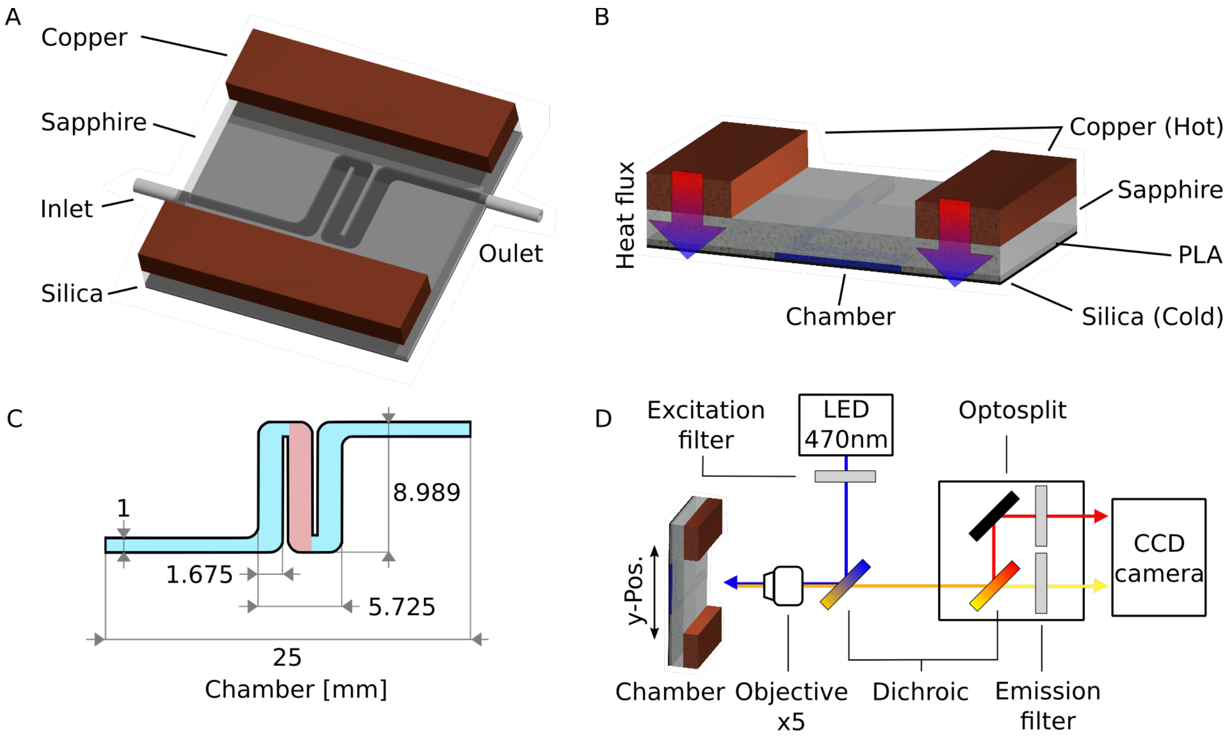

Supplementary Figure 1 | Experimental set-up for the generation and detection of pH gradients across water filled compartments. (A, B) Schematics of the experimental device. An S-shaped flow chamber is sandwiched between a transparent sapphire and a silica wafer. The copper/sapphire is heated up by an PID-controlled resitor while simultaneously cooling the silica wafer by a water bath. As a result, a fully tunable temperature gradient is formed across the flow chamber. (C) The boundaries of the flow chamber are fabricated by a 3-D printer using polylactic acid (PLA) material. The inner dimensions of the chamber (blue, red) are reduced from 1.675 x 0.2 x 8.989 to 1.2 x 0.17 x 8.0 mm during the enclosing process, where the sapphire windows is placed on top of the printed structure at 150°C for approx. 10 min. The pH is measured in the marked area of the chamber (red). (D) The ratiometric detection of the pH is achieved by a fluorescence microscope equipped with an optosplit. The y-position of the chamber can be shifted by a translation stage to simultaneously detect pH changes along the height of the chamber.

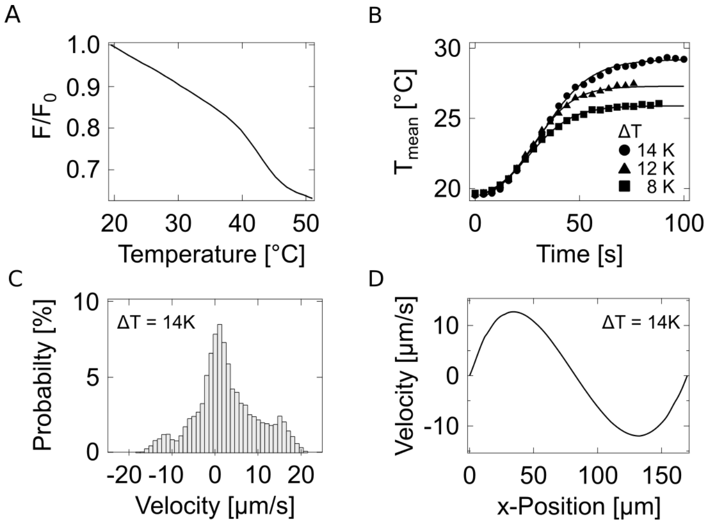

Supplementary Figure 2 | Calibration of the temperature gradient across the flow chamber. (A) Reference measurement for the temperature dependent fluorescence of (2',7'-Bis-(2-Carboxyethyl)-5-(and-6)-Carboxyfluorescein) (BCECF, 50 µM) diluted in TRIS buffer (10 mM). (B) The thermal equilibrium of a flow chamber was reached after maximum 90 s, exhibiting a mean temperature of 25.9 °C, 27.3 °C and 29.2 °C by applying a temperature gradient of ΔT = 8, 12 and 14 K, respectively. (C) The convection speeds inside a flow chamber were measured by using fluorescently labeled beads in a thermal gradient of ΔT = 14 K. (D) The experimental convection speeds are in good agreement with a 2-D finite-element simulations.

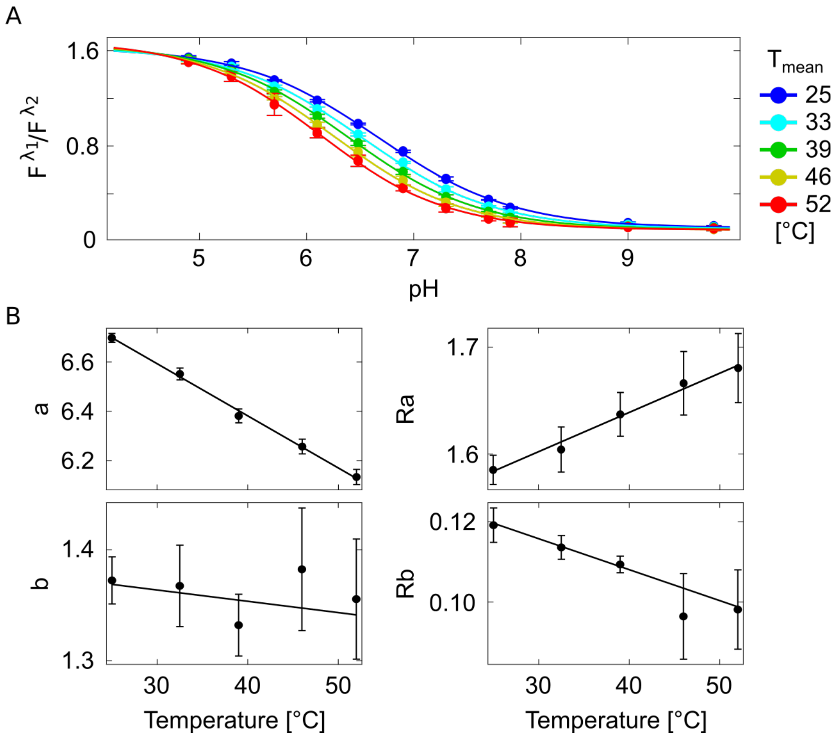

Supplementary Figure 3 | pH calibration curve for SNARF-1. (A) The fluorescence intensity ratio $R = F^{\lambda1}/F^{\lambda2}$ was measured for acidic and basic pH values in the range of pH 4.9 to 9.8 at different temperatures. $F^{\lambda1}$ and $F^{\lambda2}$ denote the fluorescence intensity of SNARF-1 at $\lambda1 = 580$ nm and $\lambda1 = 640$ nm. A modified Henderson-Hasselbalch equation (see Equation 9) comprising four parameters (*Ra, Rb, a, b*) was fitted to the ratio-to-pH calibration curve. The parameters *Ra* and *Rb* account for the maximum and minimum fluorescence rations while *a* and *b* denote for the pK_a of SNARF-1 and the weighting of the spectra. The pH calibration curve shifts with respect to the mean temperature. (B) The pH calibration curve was adjusted to the mean temperature of each measurement. Hereby, the parameters *a, b, Ra, Rb* are fitted linearly. The error bars depicts the standard deviation of the SNARF-1 fluorescence.

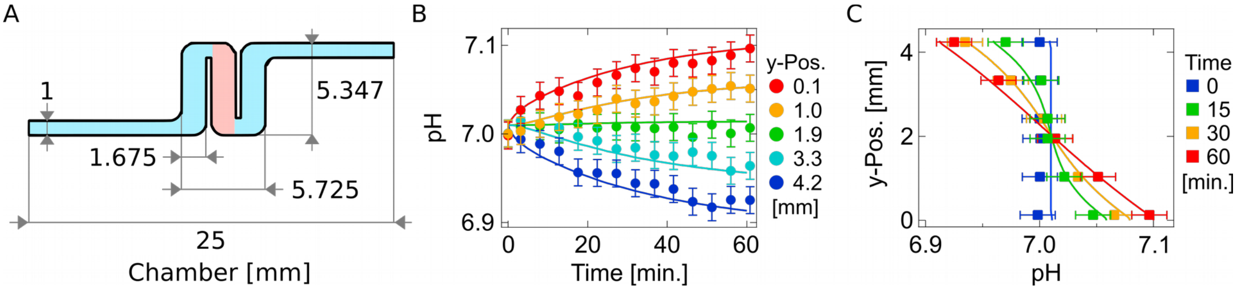

Supplementary Figure 4 | pH gradient in a shortened flow chamber. (A) Dimensions of the shortened flow chamber in mm. Reducing the height of the chamber from 8.9 to 5.347 mm results in an effective height of 4.3 mm in the measurement area (red) after the enclosure step (see Supplementary Figure 1). (B, C) The height of the flow chamber increases the equilibration time and the steepness of the proton gradient. By reducing the height of the flow chamber, the pH gradient of a phosphate buffer solution decreased from ΔpH = 0.47 to ΔpH = 0.17 after 60 min (see Supplementary Figure 8). However, the shortened chamber reached its equilibrium after 60 min, approximately four times faster compared to the 8 mm flow chamber. Finite-element simulations are in good agreement with experimental data. The error bars depicts the standard deviation of the SNARF-1 detection method.

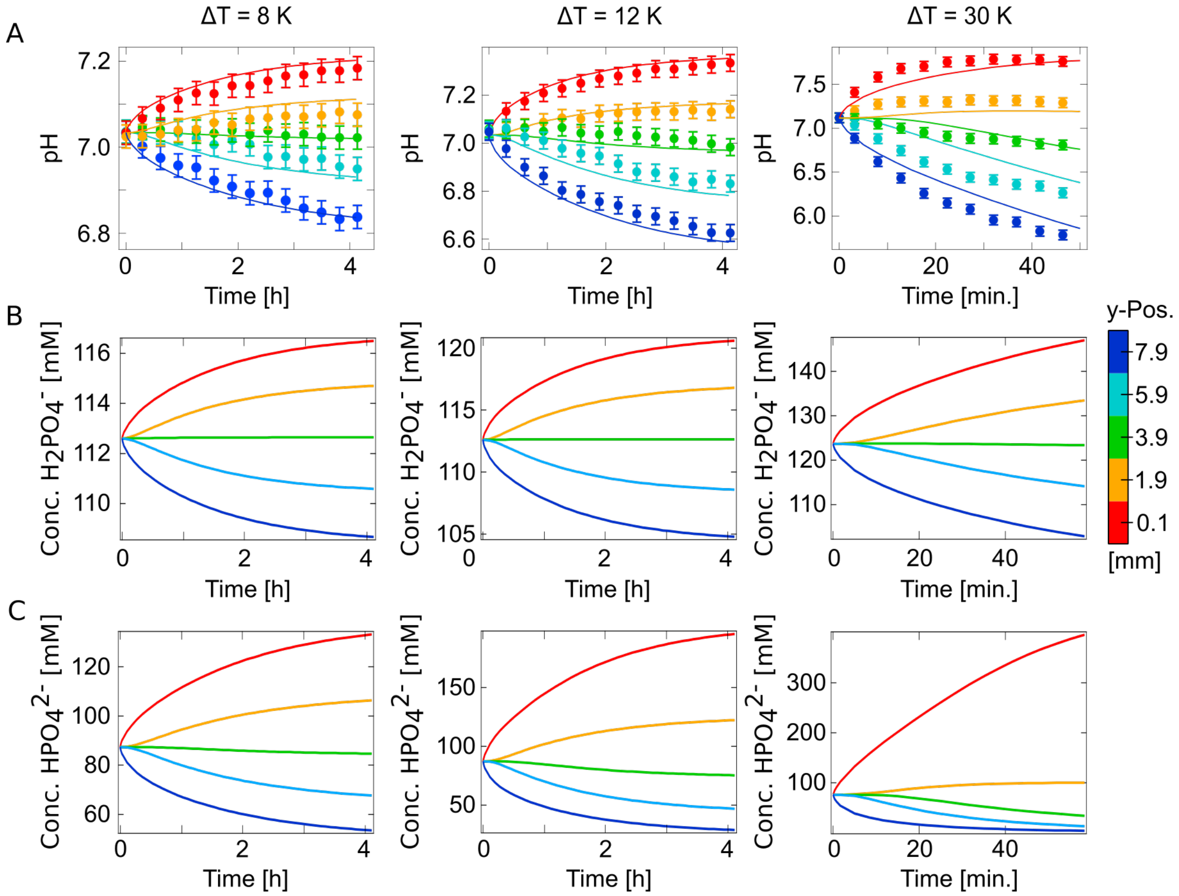

Supplementary Figure 5 | pH gradient increases with temperature difference. (A) The pH gradient was measured for temperature differences of ΔT = 8, 12, 30 K in 200 mM phosphate buffer, reaching an average temperature of 25.9, 27.3, 37.1°C, respectively. The maximum pH gradient was formed between the upper and lower regions of the chamber and increases with the steepness of the thermal gradient. The formation of a pH gradient is modulated by a finite element simulation and fits well the experimental data. (B, C) *In silico* accumulation of hydrogen phosphate and dihydrogen phosphate at specific positions in the chamber. Hydrogen phosphate accumulates more strongly at the bottom of the chamber compared to dihydrogen phosphate due to its higher Soret coefficient. As a result, a conjugate acid, dihydrogen phosphate, is formed from the reception of a proton by hydrogen phosphate. The protonation reaction locally reduces the oxonium ion concentration and therefore increase the pH at the bottom of the chamber. Phosphate and *ortho*-phosphoric acid are also included in the simulation, however the concentration of both molecules is 10^4-times lower and are therefore not depicted. The error bars depicts the standard deviation of the SNARF-1 detection method.

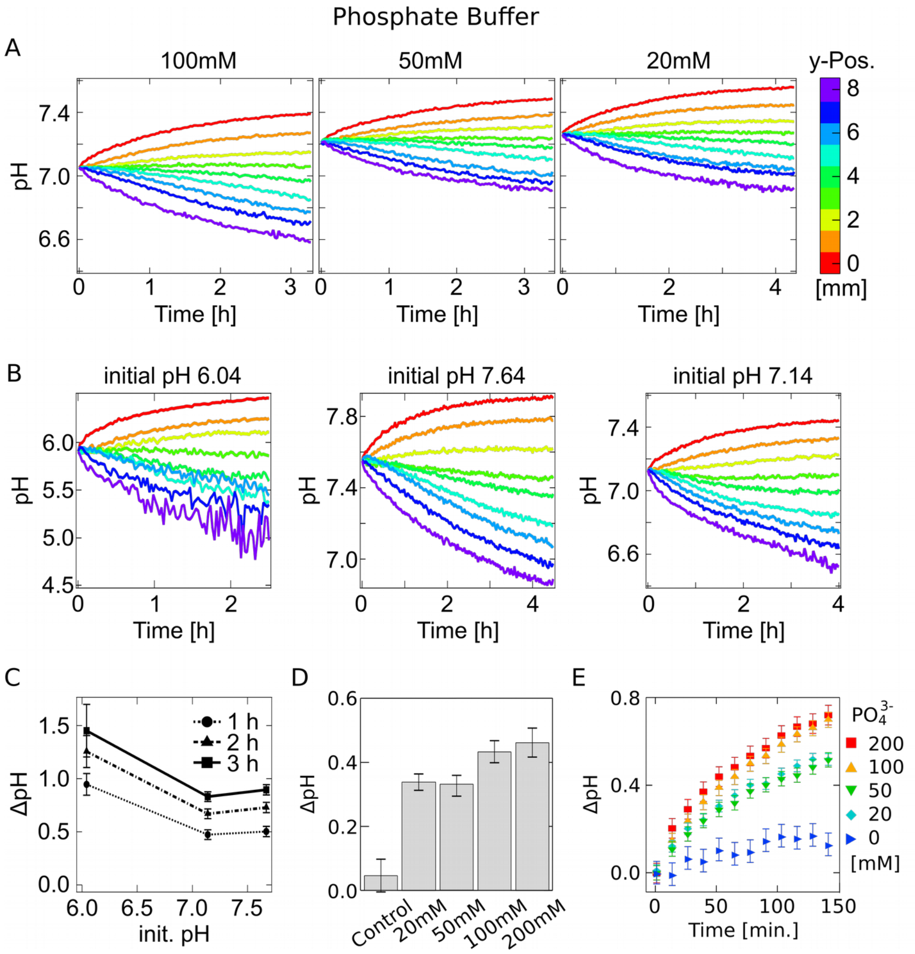

Supplementary Figure 6 | Effects of phosphate buffer concentration and initial pH on the pH gradient. (A) The pH gradient slightly increases with the phosphate buffer concentration, shown for 20, 50 and 100 mM for different y-positions in the chamber. (B, C) The initial pH affects the pH gradient, achieving the highest pH gradient for a low initial pH. Large pH fluctuation during the pH 6.04 measurement can be attributed to the detection limit of SNARF-1 from pH 5 to 9. (D) A pH gradient of 0.47, 0.44, 0.34, 0.33 and 0.05 was formed for phosphate buffer concentrations of 100, 50, 20 and 0 mM after 60 min. (E) The maximum pH gradient increases for longer experimental times. The error bars depict the standard deviation of the SNARF-1 detection method.

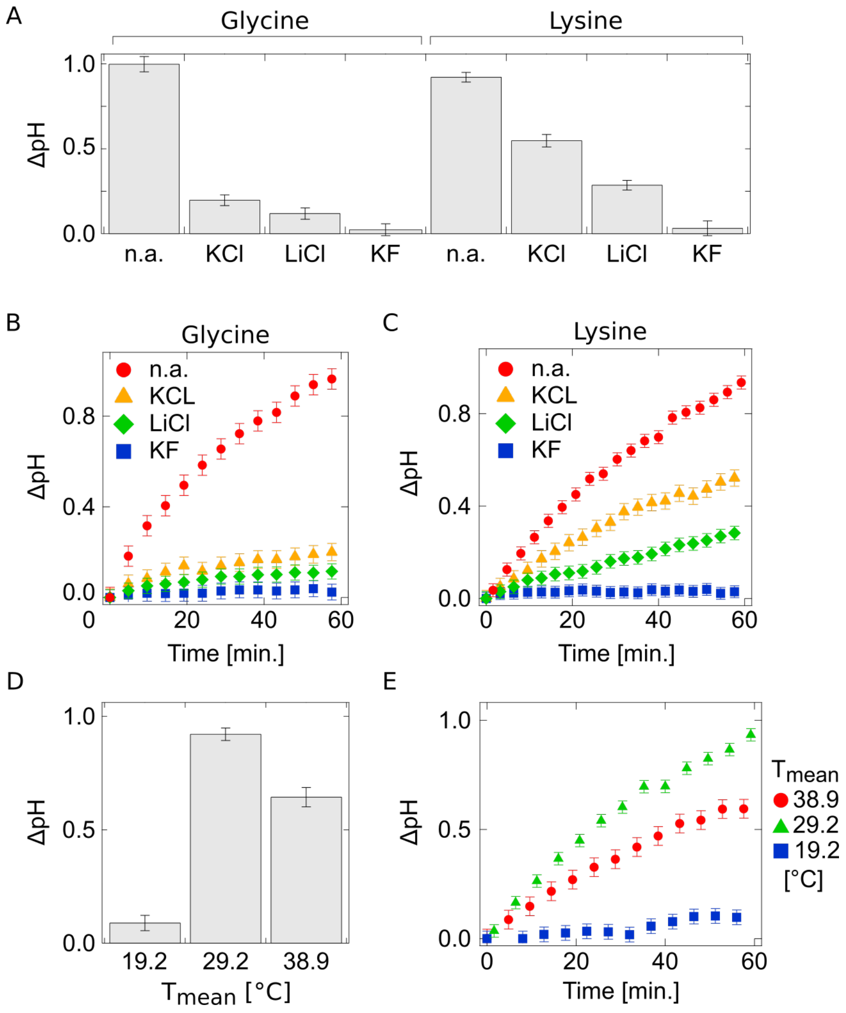

Supplementary Figure 7 | Ionic composition and temperature affect the formation of pH gradients. (A - C) The Seebeck contribution S_T^{EL} (see Equation 12) affects the formation of the pH gradient. In accordance with Reichel et al. [1], the addition of the salt species LiCl or KF decrease the Seebeck contribution S_T^{EL} while the addition of KCL results in an increase of S_T^{EL}. The overall lower pH gradient can be attributed to the high ionic strength, which decreases the Debye length and therefore the accumulation efficiency. The measurements were performed using 50 µm SNARF-1, 200 mM phosphate buffer, 500 mM salt concentrations and a temperature gradient of 14 K. (D, E) The pH gradient can also be modulated by the overall temperature based on the temperature dependence of the Soret coefficients.[1,2] The maximum pH gradient was formed for a mean temperature of 29.2 °C while the pH gradient decreases for higher and lower temperatures. The pH gradient strongly increases between T_{mean} = 19.2 °C and 29.2 °C based on the temperature dependence of the Soret coefficient S_T. The decrease in ΔpH for T_{mean} = 38.9 °C can be attributed to the accumulation behavior of the thermal trap. Here, the accumulation prefactor (see Equation 10, 11) strongly decrease since the width of the thermal trap is specifically designed for a certain diffusion coefficient (see Supplementary Figure 12).

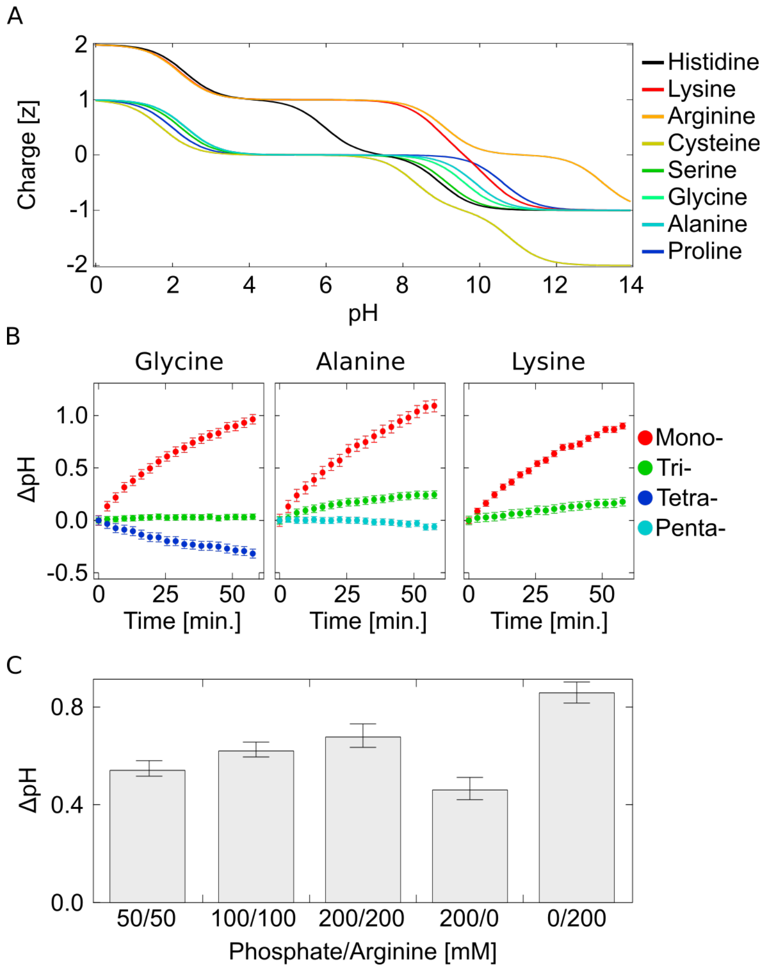

Supplementary Figure 8 | Formation of pH gradients with respect to charge, peptide length and amino acid/buffer mixtures. (A) The net charge of amino acids decreases with respect to the surrounding pH as a result of the alpha-amino group ($pK \approx 9$), the carboxylic acid group ($pK \approx 2$) and the side chain (see Equation 15). Since the initial pH was adjusted to be situated in the detection range of SNARF-1 (pH 5-9), solely the dissociation of the alpha-amino group and side-chain are detectable. (B) The formation of a pH gradient depends on the length of the peptide. For Alanine, Glycine and Lysine, single amino acid achieved the highest pH gradient, while Tetra- and Pentapetides resulted in an inversed pH gradient. An inversion of the pH gradient can be attributed to negative difference in Soret coefficient $\Delta S_T = S_{T\ acceptor} - S_{T\ donor}$. (C) The mixture of amino acid and phosphate buffer strongly affects the formation of pH gradients. Single-component buffers of phosphate and arginine formed a pH gradient of ΔpH = 0.47 and 0.86, respectively, whereas a combination of both buffers formed intermediate pH gradients. The decrease in ΔpH for 200 mM of the two component mixture can be attributed to an increase in Debye length, which in turn results in a decrease in S_T. The error bars depicts the standard deviation of the SNARF-1 detection method.

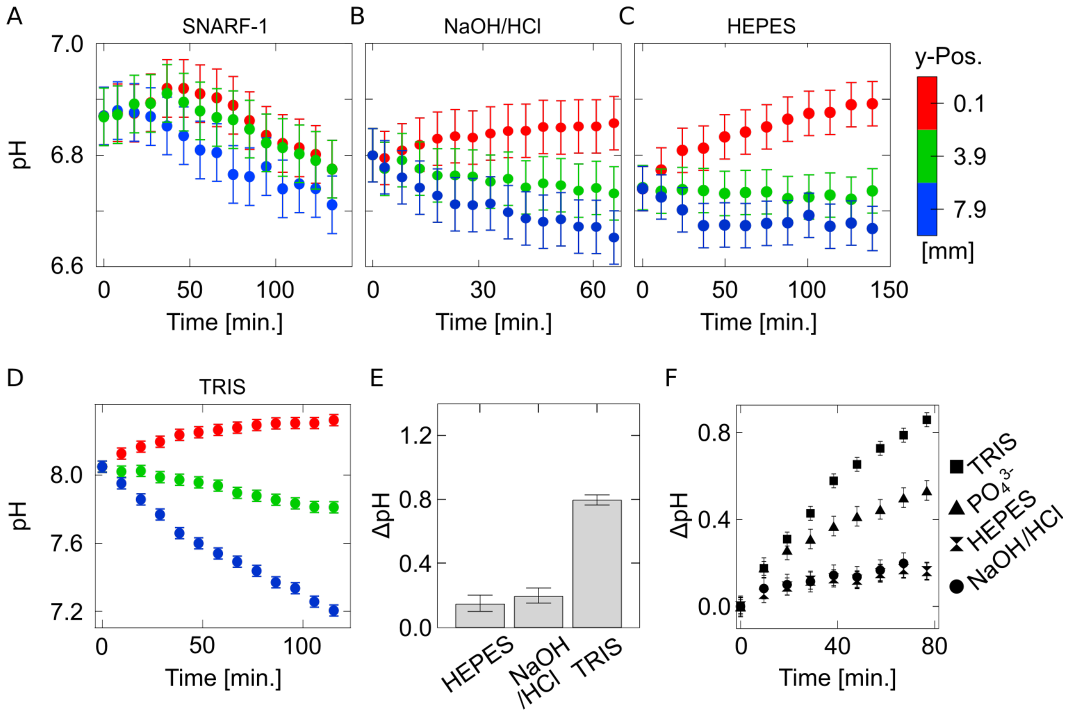

Supplementary Figure 9 | Effects of buffers and ions on the pH gradient. (A) The pH indicator SNARF-1 also acts as a buffer ($pK \approx 7.5$), forming a maximum pH gradient of $\Delta pH = 0.12$ for 50 µM SNARF-1 concentration. This concentration is used for all pH measurements. (B) The addition of 0.2 M NaOH/HCl resulted in an increase in the pH gradient. (C, D) The magnitude of the pH difference depends on the buffer. HEPES formed a shallow pH gradient while TRIS formed a relative steep pH gradient, both at a concentration of 0.2 M. (E, F) A pH gradient of $\Delta pH = 0.14$, 0.19 and 0.79 was formed for HEPES, NaCl/HCl and TRIS after 60 minutes, respectively. The error bars depicts the standard deviation of the SNARF-1 detection method.

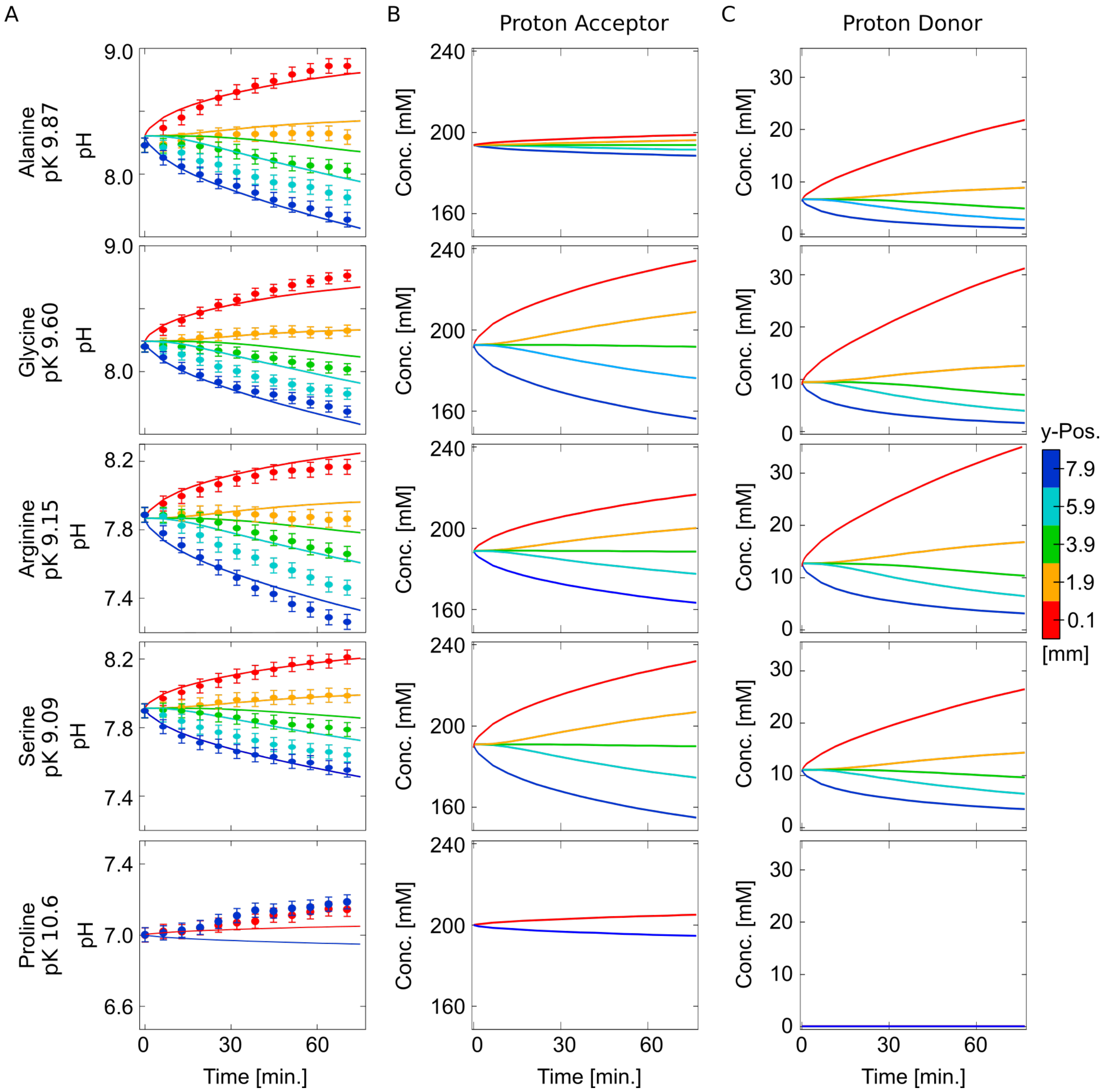

Supplementary Figure 10 | Formation of pH gradients for prebiotic plausible amino acids. (A) The pH gradient was measured for different amino acids with respect to the y-position in the chamber. The measurements were performed at an amino acid concentration of 200 mM. The starting pH was adjusted to be situated close to the pK of the amine group to ensure a sufficient quantity of both molecules, proton acceptor and donor. Finite-element simulation reproduce the increase in pH gradient by assuming a $\Delta S_T = S_{Tacceptor} - S_{Tdonor}$ of 0.022, 0.019, 0.016, 0.012 and 0.003 K^{-1} for alanine, glycine, arginine, serine and proline, respectively. (B, C) Both species, proton acceptor and donor, exhibit an increase in concentration at the bottom and a depletion at the top of the chamber. However, the accumulation of the proton acceptor is much stronger compared to the proton donor. The error bars depicts the standard deviation of the SNARF-1 detection method.

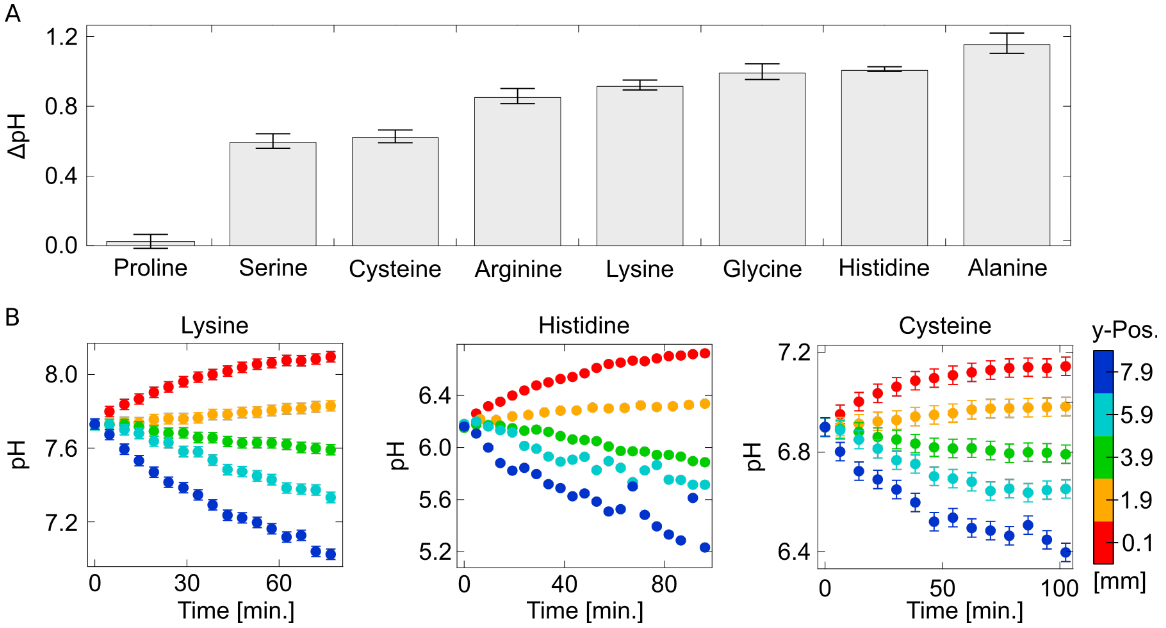

Supplementary Figure 11 | pH gradients for various amino acids in a thermal gradient. (A) The magnitude of the pH gradient strongly depends on the amino acid. After 60 min, alanine formed the highest pH gradient of $\Delta pH = 1.16$ compared to the lowest $\Delta pH = 0.03$ formed by proline. The measurements were performed at an amino acid concentration of 200 mM and 50 µM SNARF-1. (B) The pH was measured for lysine, histidine and cysteine with respect to the y-position in the chamber. In the course of the experiment, the pH decreased in the lower regions and increased in the upper regions of the chamber. The error bars depicts the standard deviation of the SNARF-1 detection method.

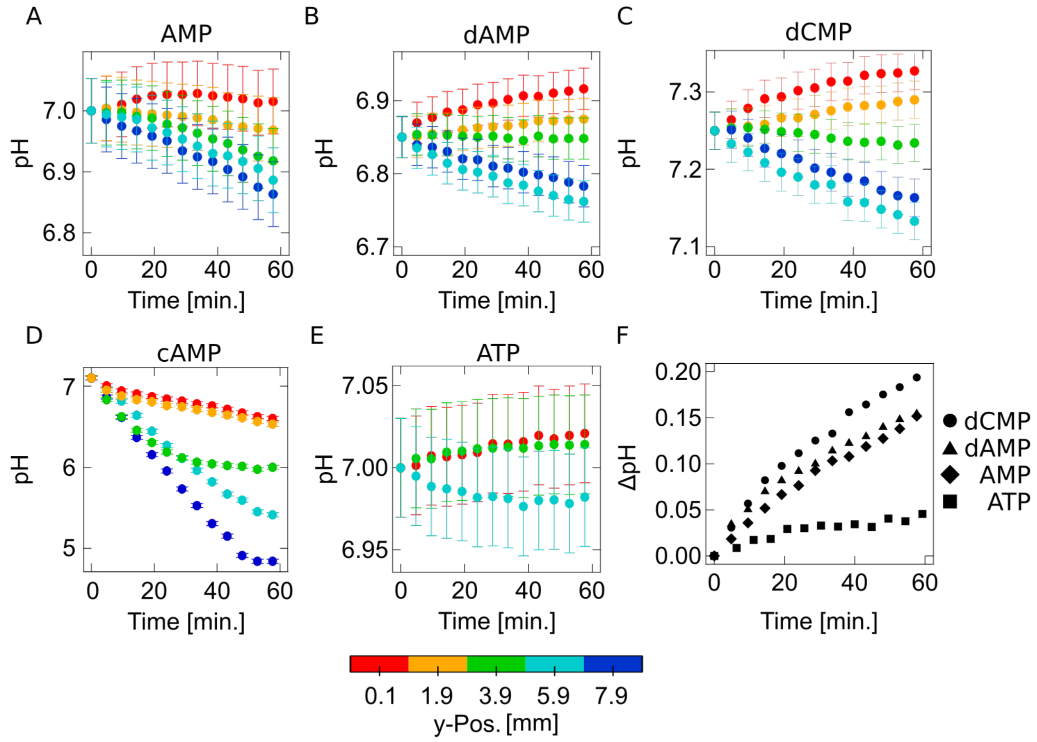

Supplementary Figure 12 | Formation of a pH gradients in solutions of RNA/DNA nucleotides. (A-E) Single nucleotides in solution with a concentration of 200 mM formed pH gradients of up to ΔpH = 0.2 within 60 min. The lowest pH gradient was achieved for ATP, having a pH gradient of ΔpH = 0.05. In the case of cAMP, a global shift in pH of several units was measured that cannot be described by our standard accumulation model. (F) dAMP, AMP and dCMP formed comparable pH gradients between upper and lower regions of the pore. The error bars give the standard deviation of the SNARF-1 detection.

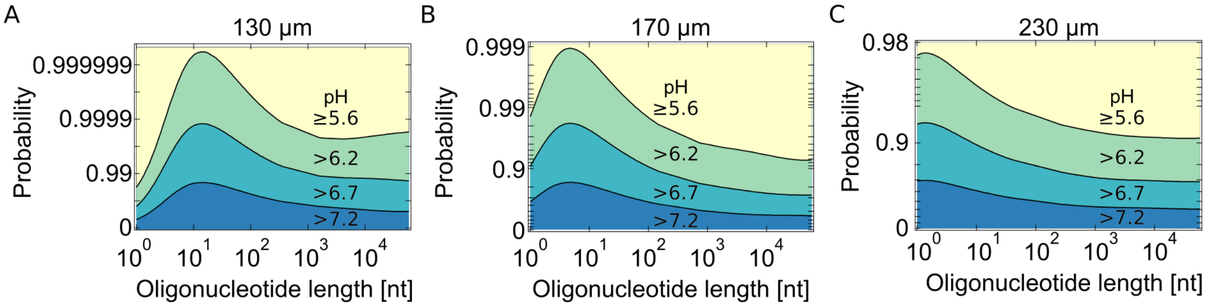

Supplementary Figure 13 | Accumulation kinetics of DNA/RNA strands depends on flow chamber thickness. (A) Within the considered experimental and simulation timescale (3h), a flow chamber with a thickness of 130 µm strongly accumulates larger molecules such as long DNA/RNA strands at the bottom, while the concentration distribution along the y-axis of short molecules is mostly given by Brownian motion. (B, C) Short molecules accumulate more efficiently in wider chambers such as 170 µm and 230 µm. Here, high convection speeds can counterbalance the fast diffusion of small molecules. The flow chamber can be specifically adjusted that certain molecules remain at defined pH regions or undergo frequent pH oscillations.

Supplementary Tables

Temp. [K]	pK_w
273.13	14.943
298.13	13.997
333.13	13.262

Supplementary Table 1 | Temperature dependence of pK_w.[3]

Variable	Formula	D [µm²/s]	S_T 10^{-3}[1/K]	pK_a	µ [10^{-7} m²/(V*s)]
Phosphate	PO_4^{3-}	824	24.0	2.12	-2.89
Hydrogen phosphate	HPO_4^{2-}	760	12.0	2.12; 7.21	-1.18
Dihydrogen phosphate	$H_2PO_4^-$	959	0.7	7.21; 12.67	-0.373
ortho-Phosphoric acid	H_3PO_4	959	0.3	12.67	
Oxonium ion	H_3O^+	9311	18.0		3.62
Hydroxide ion	OH^-	5273	23.3		-2.05
Sodium ion	Na^+	1334	8.7		0.519

Supplementary Table 2 | Input parameters for finite-element simulation. The Soret coefficients for the oxonium ion, hydroxide ion and sodium ion are taken from literature,[4,5] while the Soret coefficients of the phosphate buffer are estimated with respect to their net charge. The diffusion coefficients and mobilities are taken from Lide et al.[6]

Variable	Concentration [mol/l]
$c(H_3PO_4)$	$c(H_2PO_4^-) \cdot 10^{-pH+pK1}$
$c(H_2PO_4^-)$	$c(HPO_4^{2-}) \cdot 10^{-pH+pK2}$
$c(HPO_4^{2-})$	$c(PO_4^{3-}) \cdot 10^{-pH+pK3}$
$c(PO_4^{3-})$	$c0/(1+10^{-pH+pK3})$
c_0	$200 \cdot 10^{-3}$
$c(HX)$	$c(X^-) \cdot 10^{-pH+pKa}$
$c(X^-)$	$c0/(1+10^{-pH+pKa})$

Supplementary Table 3 | Initial concentration for the phosphate buffer and amino acids.

	pK_a
pK1	2.12
pK2	7.21
pK3	12.67

Supplementary Table 4 | pK_a values of phosphate buffer.

Variable	Rate constant
k_{offP1} [l/(mol*s)]	10^{13}
k_{offP2} [l/(mol*s)]	10^{13}
k_{offP3} [l/(mol*s)]	10^{13}
K_{si} [mol/l]	10^{-pKi}
k_{onP1} [1/s]	$k_{offP1} \cdot K_{S1}$
k_{onP2} [1/s]	$k_{offP2} \cdot K_{S2}$
k_{onP3} [1/s]	$k_{offP3} \cdot K_{S3}$
k_A [l/(mol*s)]	$1.3 \cdot 10^{10}$
K_W [mol/l]	10^{-pKw}
k_D [1/s]	$k_A \cdot Kw$

Supplementary Table 5 | Reaction rates of the phosphate buffer and water. The pKi denotes the pK_a of a specific buffer species.[7]

Rate/Conc.	$c(H_3O^+)$	$c(OH^{-})$	$c(PO_4^{3-})$	$c(HPO_4^{2-})$	$c(H_2PO_4^-)$	$c(H_3PO_4)$	$c(H_20)$
$d(H_3O^+)/dt$		$- k_A \cdot cH$	$- k_{offP3} \cdot cH$	$k_{onP3} - k_{offP2} \cdot cH$	$K_{onP2} - k_{offP1} \cdot cH$	k_{onP1}	k_D
$d(OH^-)/dt$	$- k_A \cdot cH$						k_D
$d(PO_4^{3-})/dt$			$- k_{offP3} \cdot cH$	k_{onP3}			
$d(HPO_4^{2-})/dt$			$k_{offP3} \cdot cH$	$- k_{offP2} \cdot cH - k_{onP3}$	k_{onP2}		
$d(H_2PO_4^-)/dt$				$k_{offP2} \cdot cH$	$- k_{offP1} \cdot cH - k_{onP2}$	k_{onP1}	
$d(H_3PO_4)/dt$					$k_{offP1} \cdot cH$	$- k_{onP1}$	

Supplementary Table 6 | Reaction kinetics of phosphate buffer.

Aminoacid	Initial pH	pK_a	$S_{T\ acceptor}$ [$10^{-3}\cdot 1/K$]	$S_{T\ donor}$ [$10^{-3}\cdot 1/K$]	ΔS_T [$10^{-3}\cdot 1/K$]
Alanine	8.3	9.87	22.0	0.1	21.9
Glycine	8.2	9.60	22.0	3.0	19.0
Argenine	7,9	9.15	18.0	2.0	16.0
Serine	7.9	9.09	15.0	3.0	12.0
Proline	7.0	10.6	3.0	0.1	2.9

Supplementary Table 7 | Soret coefficients, pK_a and initial pH of amino acids. The initial pH is adjusted to be situated in the SNARF-1 detection range of $5 \leq pH \leq 9$. The diffusion coefficients for amino acids are set to 760 and 960 $\mu m^2/s$ for the proton donor and acceptor, respectively.[8] The difference in Soret coefficient ΔS_T is hereby defined by $\Delta S_T = S_{T\_acceptor} - S_{T\_donor}$.

Variable	Rate constant
k_{offAA} [l/(mol*s)]	10^{13}
K_{AA} [mol/l]	10^{-pKa}
k_{onAA} [1/s]	$k_{offAA}\cdot K_{AA}$

Supplementary Table 8 | Reaction rates of amino acids.

Rate/Conc.	$c(H_3O^+)$	$c(OH^-)$	$c(X^-)$	$c(HX)$	$c(H_20)$
$d(H_3O^+)/dt$		$- k_A\cdot cH$	$- k_{offAA}\cdot cH$	K_{onAA}	k_D
$d(OH^-)/dt$	$- k_A\cdot cH$				k_D
$d(X^-)/dt$			$- k_{offAA}\cdot cH$	k_{onAA}	
$d(HX)/dt$			$+ k_{offAA}\cdot cH$	$-k_{onAA}$	

Supplementary Table 9 | Reaction kinetics of amino acids. Hereby X^- and HX denote the proton acceptor and donor, respectively.

Parameter	Offset t	Slope m [1/°C]
a	7.2293 ± 0.0407	-0.021194 ± 0.00113
b	1.3941 ± 0.0564	-0.0010164 ± 0.00165
Ra	1.4917 ± 0.0351	0.0036805 ± 0.00102
Rb	0.13895 ± 0.00986	-0.00077193 ± 0.000272

Supplementary Table 10 | Temperature dependence of SNARF-1 calibration curve. The temperature dependent correction of the SNARF-1 parameters is fitted linearly: $y = t + m \cdot x$.

Supplementary References

1. Reichl, M., Herzog, M., Götz, A. & Braun, D. Why Charged Molecules Move Across a Temperature Gradient: The Role of Electric Fields. *Phys. Rev. Lett.* **112,** 198101 (2014).
2. Iacopini, S., Rusconi, R. & Piazza, R. The “macromolecular tourist": Universal temperature dependence of thermal diffusion in aqueous colloidal suspensions. *Eur. Phys. J. E* **19,** 59–67 (2006).
3. Harned, H. S. & Owen, B. B. *The Physical Chemistry of Electrolytic Solutions* (Reinhold, New York, 1958).
4. Takeyama, N. & Nakashima, K. Proportionality of intrinsic heat of transport to standard entropy of hydration for aqueous ions. *J. Solution Chem.* **17,** 305–325 (1988).
5. Petit, C. J., Hwang, M.-H. & Lin, J.-L. The Soret effect in dilute aqueous alkaline earth and nickel chloride solutions at 25°C. *Int. J. Thermophys.* **7,** 687–697 (1986).
6. Lide, D. R. *CRC handbook of chemistry and physics.* 82nd ed. (CRC Press, Boca Raton, 2001).
7. Tinoco, I. *Physical chemistry. Principles and applications in biological sciences.* 5th ed. (Pearson, Boston, 2014).
8. Ma, Y., Zhu, C., Ma, P. & Yu, K. T. Studies on the Diffusion Coefficients of Amino Acids in Aqueous Solutions. *J. Chem. Eng. Data* **50,** 1192–1196 (2005).
