# Supplementary material for: Proton gradients and pH oscillations emerge from heat flow at the microscale
Source: Nat Commun. 2017 Dec 1;8:1897. doi: 10.1038/s41467-017-02065-3 (PMC5711904; doi:10.1038/s41467-017-02065-3)
Supplement: Supplementary file 3 — Description of Additional Supplementary Files [file 41467_2017_2065_MOESM3_ESM.pdf]

Supplementary Movies

Mast_M1 | Supplementary Movie 1

Formation of a stable pH gradient in a phosphate buffer solution. A temperature difference of $\Delta T = 14$ K across a confined 200 mM phosphate buffer solution forms a pH gradient of up to $\Delta pH = 1.1$. Hereby, hydrogenphosphate accumulates much stronger compared to dihydrogen phosphate, locally shifting the acid-base equilibrium. As a result, a pH gradient is formed in the order of several hours.

Mast_M2 | Supplementary Movie 2

Qualitative visualization of particle trajectories inside the flow chamber. The field of view is pointed at the middle of the flow chamber at a height of 4 mm. The polystyrene beads with a diameter of 1 µm are shuttled in the convective flow up- and downwards. As the beads shuttle in the chamber, their surrounding medium changes its pH due to the chamber`s accumulation characteristics. As a results, molecules that remain inside the convective flow undergo frequent pH oscillations with cycle times in the order of several minutes.
